# Promoting Patient and Caregiver Engagement in Self-Management of Chronic Illness

**DOI:** 10.1155/2013/180757

**Published:** 2013-05-28

**Authors:** Annette DeVito Dabbs, Mi-Kyung Song, Sabina De Geest, Patricia M. Davidson

**Affiliations:** ^1^School of Nursing, University of Pittsburgh, Pittsburgh, PA, USA; ^2^School of Nursing, UNC-Chapel Hill, Chapel Hill, NC, USA; ^3^Institute of Nursing Science of the Faculty of Medicine at the University of Basel, Switzerland; ^4^Faculty of Health, University of Technology, Sydney, Australia

As a nursing professional you are no doubt aware of the growing prevalence of people with chronic conditions and the problems they face interacting and getting support from health care systems that are designed to deal with acute problems. Chronic diseases, such as heart disease, stroke, cancer, respiratory diseases and diabetes, are by far the leading cause of morbidity and mortality in the world [1]. Furthermore, more than half of individuals with one chronic condition have multiple chronic conditions, increasing the complexity and burden of managing their health. 

Chronic conditions require a life-long care perspective. Support for patient's self-management is an essential component in the care of the chronically ill in order to guarantee favorable outcomes. Self-management has been defined as the individual's ability to manage symptoms, treatment, physical and psychosocial consequences and lifestyle changes inherent in living with a chronic condition [2]. 

Patients and families are often on the front-line managing chronic illness between formal contacts with their healthcare providers. Effective self-management of chronic conditions requires patients and families to be engaged in their care in the face of dynamic changes in disease condition, complexity, symptoms, burdens, support and coping resources. Certain events and points of transition arise over the course of illness and make patients and families more vulnerable when their ability to perform self-management and maintain their quality of life is threatened. Due to the dynamic nature of chronic illness, patients' and families' needs for skills, information and support vary at different points in time, environments and situations. Because of the dynamic realities of living with a chronic condition, they need a repertoire of self-management skills to deal with the unpredictable and sudden variations. 

All of the papers in this special issue inform the science of self-management. Several authors employed integrated review and meta-analytic techniques, including Altman Klein and colleagues whose review identified the need for more responsive and dynamic education interventions for self-management of diabetes; G. S. Rasmussen and colleagues' integrative review of the problems and vulnerability associated with having psoriasis during adolescence; the study conducted by E. Kendall and colleagues points to the need for a more ecological model for the management of illness that actively engages consumers in social relationships and addresses the context within which their lives (and illnesses) are enacted; and the mapping of the literature regarding patient engagement conducted by S. Barello and colleagues which revealed several emerging challenges to promoting patient engagement. 

Other authors presented interventions to promote self-management in specific patient populations, including Hellström and colleagues for children with long-term illness to make a healthy transition as they enter school; J. Yang for Korean Patients with Chronic Hepatitis B; and B. L. Faett and colleagues for persons with chronic lower limb edema. 

Perhaps not surprisingly because of the complexity of the topic, many of the original research reports that were selected for publication relied on qualitative inquiry, including phenomenography, grounded theory, qualitative description, bibliometric and qualitative content analysis and interpretations of visual data of behavior and dialogue. Specifically these papers contribute to our understanding of the experiences of patients and families as they face the challenging dynamics and complexities of managing chronic conditions including periods of transition and adjustment. The work of O. Mauthner and colleagues highlights the unique experiences and perspectives of heart recipients after transplant. M. Salminen-Tuomaala and colleagues describe the impact of variations in coping resources after myocardial infarction; K. Wickersham and colleagues describe the demanding experience of medication management among women receiving Anastrozole therapy. 

In sum, the work included in this issue underscores the variety of challenges patients and families face and the importance of developing innovative interventions to assist and amplify their engagement in self-management behaviors in order to maximize quality of life and positive health outcomes.

*Annette DeVito Dabbs* *Annette DeVito Dabbs* *Mi-Kyung Song* *Mi-Kyung Song* *Sabina De  Geest* *Sabina De  Geest* *Patricia M. Davidson* *Patricia M. Davidson*
